# How to Choose the Biologic Therapy in a Bio-naïve Patient with Inflammatory Bowel Disease

**DOI:** 10.3390/jcm11030829

**Published:** 2022-02-04

**Authors:** Viviana Laredo, Carla J. Gargallo-Puyuelo, Fernando Gomollón

**Affiliations:** 1 Department of Gastroenterology, University Clinic Hospital Lozano Blesa, 50009 Zaragoza, Spain; fgomollon@gmail.com; 2 Institute for Health Research Aragón (IIS Aragón), 50009 Zaragoza, Spain; 3 Department of Medicine, Psychiatry and Dermatology, University of Zaragoza, 50009 Zaragoza, Spain; 4 Liver and Digestive Diseases Networking Biomedical Research Centre (Centro de Investigación Biomédica en Red, Enfermedades Hepáticas y Digestivas, CIBEREHD), 28029 Madrid, Spain

**Keywords:** inflammatory bowel disease, biologic therapy, bio-naïve

## Abstract

The availability of biologic therapies in inflammatory bowel disease (IBD) is increasing significantly. This represents more options to treat patients, but also more difficulties in choosing the therapies, especially in the context of bio-naïve patients. Most evidence of safety and efficacy came from clinical trials comparing biologics with placebo, with a lack of head-to-head studies. Network meta-analysis of biologics and real-world studies have been developed to solve this problem. Despite the results of these studies, there are also other important factors to consider before choosing the biologic, such as patient preferences, comorbidities, genetics, and inflammatory markers. Given that resources are limited, another important aspect is the cost of biologic therapy, since biosimilars are widely available and have been demonstrated to be effective with a significant decrease in costs. In this review, we summarize the evidence comparing biologic therapy in both Crohn´s disease (CD) and ulcerative colitis (UC) in different clinical situations. We also briefly synthesize the evidence related to predictors of biologic response, as well as the biologic use in extraintestinal manifestations and the importance of the drug-related costs.

## 1. Introduction

The expansion of therapeutic options in inflammatory bowel disease (IBD) makes the management of these chronic diseases more and more complex. However, choosing the right drug for the right patient at the right time is all but easy. The high variability of individual factors, the lack of data from head-to-head trials, and the limited generalization of clinical trial results to all populations make evidence-based decisions difficult. Hopefully, new clinical trials designs, more head-head studies, multiomics analysis, and artificial intelligence will change the landscape in the future [1,2,3], but while waiting for this new world, we think a review of currently available information will be useful for patients and clinicians.

## 2. Comparison of Biological Drugs for IBD in Bio-naïve Patients According to Indication

In this section, we will summarize the existing evidence on head-to-head comparison of biologicals in Crohn´s disease (CD) and ulcerative colitis (UC), based on data from randomized controlled trials (RCT) and real-world evidence (RWE).

### 2.1. In Crohn´s Disease

#### 2.1.1. Efficacy in Luminal CD

The SEAVUE study is the only head-to-head comparative RCT between two biologicals in CD [4]. It was a multicenter, randomized (1:1 ustekinumab (UST):adalimumab (ADA)), blinded, parallel-group, active-controlled study that included 386 biologic-naïve patients with moderate to severe CD through one year. Both drugs were highly effective without statistically significant differences at week 52 in clinical remission (65% and 61% with UST and ADA, respectively), corticosteroid-free remission, clinical response, and endoscopic remission. Treatment discontinuation was numerically lower for UST (15.2% vs. 23.6%), and safety data were similar to prior studies for both treatments. Infections were more frequent with ADA (34% vs. 40.5%), but serious infections occurred at a similar rate (2.1% and 2.6%). Several head-to-head comparative RCTs in CD are close to completion or are ongoing (brazikumab vs. ADA vs. placebo, mirikizumab vs. UST vs. placebo, risankizumab vs. UST, guselkumab vs. placebo vs. UST).

Given the scarcity of head-to-head RCTs, indirect comparisons can be useful. In 2021, a systematic review and network meta-analysis of 31 phase 2 and phase 3 RCTs including patients with moderate to severe CD (2931 biologic-naïve patients and 2479 patients with previous biologic exposure) was published. In naïve patients, combination therapy infliximab (IFX) plus azathioprine (OR 7.49, 95% CI 2.04–27.49), IFX (OR 4.53, 95% CI 1.49–13.79), ADA (OR 3.01, 95% CI 1.25–7.27), and UST (OR 2.63, 95% CI 1.10–6.28) were more effective in achieving remission than certolizumab pegol (CTZ). IFX plus azathioprine was also more effective than vedolizumab (VDZ) and CTZ. In patients with a history of prior biologic treatments, ADA, after loss of response to IFX, and risankizumab were more effective in achieving remission than VDZ. There were no differences in maintenance trials [5,6].

On the other hand, the amount of RWE that compares the effectiveness between different biologics in CD is large (Table 1) [7,8,9,10,11,12,13,14]. IFX and ADA are the most used biologic drugs in CD, and several real-world studies concluded that they appeared to have similar effectiveness in patients with CD [7,8,9,10,11,12]. The approval of VDZ and UST for CD expanded the therapeutic arsenal with biologics with a therapeutic target other than TNF alpha. There is little evidence comparing anti-TNFs with VDZ or UST in naïve to biological CD patients, but the data to date seem to indicate that there are no differences in terms of effectiveness in this indication [13,14].

#### 2.1.2. Efficacy in Fistulizing CD

Evidence on the efficacy and effectiveness of biologics is scarcer for fistulizing or penetrating CD than for luminal CD. This form of CD is difficult to treat and often requires medical plus surgical management. The efficacy of biological drugs in the subgroup of patients with penetrating CD has been poorly evaluated, and most data are extracted from subanalyses from registry studies and from small observational studies. Moreover, most of the evidence is on perianal disease, with scarce data on internal fistulae (enteroenteric, enterovesicular, enterovaginal, or enterocutaneous).

IFX is the only biological drug specifically compared against placebo in fistulizing CD. The strongest evidence on the efficacy of biologics in this scenario is with this anti-TNF drug. One positive RCT published in 1999 that showed the superiority of IFX in achieving complete fistula closure (almost all perianal) compared with placebo in 94 patients revolutionized the treatment of fistulizing CD [15]. Another RCT (ACCENT II) that evaluated IFX efficacy as maintenance treatment in 195 patients who had responded to IFX induction therapy also showed the superiority of IFX compared with placebo at week 54 (58% vs. 38%) [16]. Regarding ADA, most of the evidence comes from subanalysis of RCTs with low statistical power. Among four trials with ADA for induction, two were positive and two were negative [17,18,19,20]. A subgroup analysis of CHARM RCT that evaluated 117 patients with fistulae (almost all perianal) showed the superiority of ADA compared with placebo in fistula closure after one year of treatment (33% vs. 13%) [17,21]. There are also several real-world studies with positive results for ADA in perianal CD. Nowadays, most clinical practice guidelines consider ADA as a good option in this scenario [22,23,24]. There are no positive studies with CTZ for fistulizing CD [25,26,27]. Note that in penetrating CD, long-term treatment is frequently necessary before considering a failure of biologic. In summary, both IFX and ADA seem to be effective in this form of CD.

Data about efficacy of non-anti-TNF biologicals, VDZ and UST, in fistulizing CD are scarce. In a subanalysis of the GEMINI trial that included 165 CD patients with at least one draining fistula, VDZ seems to be superior compared to placebo for fistula remission at week 14 (23% patients with VDZ, 8 weeks; 41% patients with VDZ, 4 weeks; and 11% in the placebo group) [28]. An RCT (phase 4) that compared two regimens of VDZ (with or without an extra dose at week 10) reported that over half of patients had reductions of ≥50% in the number of draining perianal fistulae with no differences between the two doses [29]. Moreover, there is an ongoing clinical trial evaluating VDZ in this scenario (NCT02630966). Regarding UST, Sands and colleagues published a pooled analysis of four induction RCTs that included 238 patients with active perianal disease, and their analysis showed that UST was superior to placebo in reaching fistula remission [30]. In an extension of the IM-UNITI maintenance trial in which patients who had responded to UST induction were randomized to UST vs. placebo, UST was more effective (fistula closure at week 44, 80% (12/15) vs. 45.5% (5/11)) [31]. Moreover, several small, open-label case series have reported a symptomatic response rate of up 60–70% of patients with UST [32,33,34].

#### 2.1.3. Safety

Serious infections, particularly opportunistic, and malignancies are the main safety issues with the use of biologics in IBD. We will summarize key data.

##### Serious Infections

Active disease and concomitant use of steroids and/or immunomodulators are the most important risk factors for serious infections in IBD patients. Because of that, the safety of a biologic depends mainly on two factors: (1) its intrinsic immunosuppressive effect and (2) its ability to reduce inflammation, reducing the risk of IBD complications and the use of corticosteroids.

The largest amount of evidence and the highest-quality evidence on biologic drug safety have been accumulated for anti-TNFs. Registry studies and RWE have suggested that anti-TNFs may double the risk of serious infections compared with other immunomodulators [35,36,37,38]. A recent meta-analysis reported that the highest risk of serious infections was associated with combination therapy (anti-TNF plus immunomodulators) and the lowest risk was associated with immunomodulator monotherapy (combination therapy vs. anti-TNF alone: RR 1.19, 95% CI 1.03–1.37; anti-TNF vs. immunomodulators: RR 1.64, 95% CI 1.19–2.27) [39]. Interestingly, a retrospective Medicare and Medicaid cohort study reported that the risk of serious infections with anti-TNF and with long-term steroid treatment was similar, but a higher mortality with steroids was observed [40].

Regarding VDZ, in the pivotal GEMINI studies, VDZ was not associated with an increased risk of infections compared to placebo, except for an apparently higher risk of *Clostridium difficile* [41]. VDZ seems to be associated with less serious infections than anti-TNF, at least in some studies, with others showing similar data [42,43].

Registry studies and large real-world observational studies of UST in CD are awaited. A recent safety analysis of a pooled IBD population (2574 patients with CD and 1733 with UC) of six phase 2/3 trials of UST reported that the safety profile of UST was similar to placebo after one year of treatment [44]. These data are in agreement with other analyses that include cross-indications [45], longer-term analysis in psoriasis (5 years) [46], or analysis in UC [47], suggesting that serious infections risk with UST may be lower than that with anti-TNF. It should be noted that the UST dose used in psoriasis is lower than the dose used in CD, so psoriasis data should be interpreted with caution.

##### Malignancies

Long-term population studies did not report an association between anti-TNFs and the risk of solid neoplasms [48,49]. However, these drugs have been variably associated with a 2–5-fold increased risk of lymphoma [36,50,51]. A meta-analysis of four high-quality observational studies reported a similar risk of lymphoma with anti-TNF monotherapy and thiopurine monotherapy [52]. It is important to note that patients with combination therapy (thiopurine plus anti-TNF) seem to have a significant increase in the risk of lymphoma (up to 6-fold compared with nontreated patients and 2.5-fold compared with patients treated with thiopurines or anti-TNF monotherapy). By contrast, long-term follow-up of RCTs has not found an increased risk of solid-organ neoplasms or hematologic malignancies with anti-TNFs [53].

Pivotal VDZ studies have not notified an increased risk of solid-organ or hematologic malignancies, but long-term follow-up and RWE are lacking. Regarding UST, integrated safety analyses of phase 2/3 trials, including cross-indications, did not show an increased risk of malignancy; the risk of malignancy was comparable with that of placebo (0.4 vs. 0.2 per 100 person-years) [44].

Adding an immunomodulator to a biologic drug may increase its efficacy because it adds its own effectiveness and because it seems to decrease the immunogenicity of biological drugs. Anti-TNF drugs, particularly IFX, are more immunogenic than non-anti-TNF biologics [54,55]. Therefore, combination therapy to prevent immunogenicity in anti-TNF-treated patients (particularly in patients treated with IFX) may be particularly interesting when patients have an unfavorable pharmacokinetic profile or a history of immunogenicity to other anti-TNFs [53]. Nevertheless, combination therapy increases the risk of tumors and infections, so in patients with risk factors for infections and/or tumors and for the development of immunogenicity, non-anti-TNF biologics may be a better option.

Briefly, anti-TNF drugs may be more immunosuppressive than VDZ and UST and may have a higher risk of hematologic tumors and serious infections. The potential safety advantage of non-anti-TNF biologics over anti-TNF therapies may disappear if these non-anti-TNF drugs are used in combination with thiopurines, which increases the risk of infections and lymphomas.

### 2.2. In Ulcerative Colitis

#### 2.2.1. Efficacy in Moderate–Severe UC

- *Head-to-head comparisons of biologic drugs*

There are many randomized placebo-controlled trials assessing the efficacy of biologics in patients with moderate to severe UC (Table S1); however, there are only a few studies comparing efficacy between different biologic therapies. In the VARSITY study, patients with moderate to severe UC refractory to conventional therapy or other anti-TNFs were randomized assigned to be treated with VDZ or ADA [56]. Less than 25% of patients were previously treated with anti-TNFs. In the group of bio-naïve patients, at week 52, clinical remission and endoscopic improvement were superior in the VDZ group (34.2% vs. 24.3% and 43.1% vs. 29.5%, respectively). However, 12.6% of patients treated with VDZ and 21.8% of those treated with ADA were in steroid-free clinical remission at week 52.

Recently, the results of the histologic outcomes from the VARSITY trial have been published [57]. In the subgroup of bio-naïve, there were higher rates of histological remission at week 14 in the group of VDZ using either of the histological scores (remission rates according to Geboes index: 18.1% VDZ vs. 9.2% ADA, *p* = 0.0014, and according to RHI score: 27% VDZ vs. 19% ADA, *p* = 0.0198). The results were similar at week 52 (remission rates according Geboes index: 32.2% VDZ vs. 9.5% ADA, *p* < 0.0001, and according RHI score: 39.8% VDZ vs. 22.6% ADA, *p* < 0.0001). In a post hoc analysis, VDZ was also more effective in terms of mucosal healing (composite outcome of histologic remission and endoscopy improvement) (25.6% vs. 6.7%, *p* < 0.0001, and 30.5% vs. 14.5%, *p* < 0.0001, for VDZ vs. ADA according to Geboes and RHI scores, respectively). The superiority of VDZ has been recently confirmed in a retrospective Belgian study [58].

- *Network meta-analysis comparing biologic drugs*

Since head-to-head trials comparing biologic therapy in UC bio-naïve patients are lacking, some meta-analysis has been developed. In 2017, Singh et al. published a meta-analysis including RCTs comparing the efficacy of biologic therapy and small molecules in moderate–severe UC [59]. In the subgroup of naïve patients, compared with the other alternatives, IFX and VDZ had the highest rates of clinical remission (SUCRA for IFX 0.85, VDZ 0.82, golimumab (GOL) 0.58, tofacitinib 0.43, ADA 0.31) and mucosal healing (SUCRA for IFX 0.91, VDZ 0.81, tofacitinib 0.54, GOL 0.41, ADA 0.32). In this study, IFX was superior in inducing clinical remission to ADA (OR 2.33, 95% CI 1.17–4.64) and there were no differences when comparing it with VDZ (OR 0.96, 95% CI 0.30–3.09). Concerning safety, VDZ did not increase the risk of infections compared with placebo (OR 1.03, 95% CI 0.60–1.79), while anti-TNFs were associated with more infections with statistical significance in the case of GOL (OR 1.85, 95% CI 1.20–2.86) but without it in the case of IFX (OR 1.30, 95% CI 0.60–179) and ADA (OR 1.23, 95% CI 0.91–1.65). The risk of infections with 5 mg of tofacitinib was also increased (OR 1.75, 95% CI 1.13–2.70), and when using 10 mg, the risk was higher than that of ADA and VDZ. Based on this study, VDZ and IFX are the most useful biologics in achieving clinical remission and mucosal healing in anti-TNF-naïve patients with moderate–severe UC. In terms of safety, VDZ is the drug with less serious adverse events.

A similar meta-analysis was conducted with moderate–severe UC Japanese patients naïve to biologics [60]. During induction, compared with placebo, IFX and VDZ had the highest rates of remission (OR 2.35, CI 1.31–4.08; OR 2.32, CI 1.05–5.16, respectively), without differences in ADA therapy (OR 1.57, CI 0.82–2.92). In induction, only ADA and IFX showed a statistically significant effect in mucosal healing. In maintenance, GOL and VDZ had the highest rates of remission (OR 5.13 and 3.84, respectively) (there were no data on IFX). 

In the meta-analysis of Bonovas et al. in UC anti-TNF-naïve patients, IFX was superior to ADA in clinical response, remission, and mucosal healing (OR 2.01, 2.10, and 1.87, respectively), and it was also superior to GOL in clinical response and mucosal healing (OR 1.67 and 1.75, respectively) [61]. There were no differences in efficacy between tofacitinib and biologics.

Trigo-Vicente et al. carried out a meta-analysis including bio-naïve patients with moderate–severe UC, and the best biologic for induction in terms of clinical remission was IFX (IFX (OR 4.15), VDZ (OR 3.7), GOL (OR 3.2), tofacitinib (OR 2.2), and ADA (OR 1.9)) [62]. IFX was also significantly superior to ADA (OR 2.35). In maintenance, the best therapies for clinical remission were tofacitinib 10 mg and VDZ (OR 5.5 and 3.8, respectively). The odds ratios of IFX, ADA, and GOL were 2.7, 2.4, and 1.8, respectively. In this setting, tofacitinib was statistically superior to ADA and GOL. Moreover, in this study, the highest rates of infections were associated with tofacitinib and VDZ, without global differences in terms of serious adverse events between all the drugs included.

In the American Gastroenterological Association (AGA) Technical Review, in the induction of remission in bio-naïve moderate–severe UC, IFX was superior to ADA (OR 2.10, 95% CI 1.16–3.79) with moderate quality of evidence [63]. The rest of the comparisons between biologics and small molecules (IFX, VDZ, GOL, UST, tofacitinib) did not find a superiority of any of the drugs. It is important to note that the quality of evidence for these comparisons was low or very low. In maintenance, the authors explain that a meta-analysis is not useful since the designs of the studies are not comparable. Moreover, in most maintenance studies, there are no data about previous exposure to biologic therapy. A comparison between studies with similar designs was made (IFX–ADA–VDZ and GOL–VDZ-UST–tofacitinib) without finding differences between the drugs, but the quality of evidence was low again.

- *Comparison of biologic drugs in the real-world setting*

Although there are many studies assessing the efficacy of different biologic therapies in the real-world practice, the number of studies comparing two or more therapies is limited (Table 2). In a Danish study based on nationwide registry data of UC bio-naïve patients, authors used propensity score matching to compare efficacy and safety of ADA and IFX [64]. Twenty-four percent of patients had severe UC. In the group of ADA, the risks of hospitalization for any cause (HR 1.84, 95% CI 1.18–2.85, *p* = 0.007) and serious infection (HR 5.11, 95% CI 1.20–21.80, *p* = 0.03) were greater than those of IFX, without differences in hospitalization due to UC (*p* = 0.07) and surgery (*p* = 0.45). When comparing IFX and ADA, both in monotherapy, there were no differences in any of the outcomes; however, when comparing combination therapies (ADA + immunomodulator vs. IFX + immunomodulator), the group of ADA had more risk of UC-related hospitalization (HR 3.89, 95% CI 1.32–11.50, *p* = 0.01). In men, the rates of hospitalization were higher with ADA, without differences between ADA and IFX in women.

The retrospective EVOLVE study compared long-term (24 months) effectiveness and safety of anti-TNFs (mainly IFX, but also ADA and GOL) and VDZ in naïve IBD patients [65]. In this study, there were more patients with moderate–severe UC in the group of anti-TNFs than in the VDZ group (82.7% vs. 72.4%, *p* < 0.001); however, after an adjusted comparison, the rates of response, clinical remission, and mucosal healing were similar between groups (VDZ vs. anti-TNFs: 88.3% vs. 86.2%, *p* = 0.64; 65.9% vs. 48.6%, *p* = 0.09; 86.6% vs. 80.6%, *p* = 0.66, for each endpoint respectively). Concerning safety, after adjustment, the rate of serious adverse events was lower in the group of VDZ without differences in serious infections (HR = 0.37, CI 0.21–0.63; HR = 0.56, CI 0.21–1.51, respectively).

In a German study in the real-world setting, the effectiveness and safety of anti-TNFs and VDZ in IBD were evaluated [66]. In the group of bio-naïve UC patients, at week 26, 50.1% of those treated with VDZ and 31.5% of those treated with anti-TNFs were in clinical remission. In this subgroup of patients, the mean of the partial Mayo score was 4.8, and in those with information about baseline endoscopy score, more than half were classified as Mayo 0 or 1. The rate of adverse events related to treatment was 4.5% for VDZ and 7.5% for anti-TNFs. In the group of VDZ, all adverse events, except infections, occurred in less than 5% of patients. 

In a United States (US) study comparing VDZ with IFX in UC, after 24 months of therapy, the rates of treatment persistence were higher with VDZ than with IFX (78.5% vs. 63.5%, *p* = 0.046) [67]. More patients receiving IFX needed treatment intensification compared with VDZ (at 12 months 21.8% vs. 6.4%, *p* = 0.0008, and at 24 months 25.1% vs. 12.8%, *p* = 0.0022). In another real-world study in UC, there were no differences in terms of induction response between VDZ and IFX in bio-naïve patients (IRR 1.04, 95% CI 0.47–2.29) [68]. Data comparing UST with other biologic therapy in the real-world setting are scarce.

When there is indication for biologic therapy, the AGA recommends anti-TNFs in bio-naïve UC patients but also suggests that VDZ and tofacitinib could be used as first-line therapy, particularly in special populations [69].

#### 2.2.2. Efficacy in Acute Severe UC

In this setting, there is only enough evidence to recommend IFX. In a RCT including 45 patients with acute or moderately severe UC refractory to intravenous steroids, the rates of colectomy were lower among those receiving one dose of IFX compared with placebo (colectomy in 7/24 vs. 14/21; *p* = 0.017, OR 4.9) [70]. There are no other RCTs or prospective studies with the rest of the biologics in this setting. The alternative to IFX is cyclosporine, without differences in terms of colectomy (RR 1.00, 95% CI 0.72–1.40) [63]. When using cyclosporine as induction, a different maintenance treatment is necessary. Traditionally, thiopurines were the drugs used for maintenance; however, there is recent evidence supporting the utility of some biologics [71]. Ollech et al. conducted a retrospective study of patients with severe UC refractory to steroids and treated with calcineurin inhibitors as induction therapy and, after that, treated with VDZ for maintenance [72]. Only 15% of patients were bio-naïve. This sequential therapy avoided colectomy in 67% and 55% of patients after 12 and 24 months. Steroid-free remission rates were 27%, 43%, and 76% at 3, 12, and 24 months, respectively. The treatment with VDZ had to be intensified every 4 weeks in 44% of patients after a mean time of 5.6 months. Case reports in acute UC patients with previous exposure to anti-TNFs treated with calcineurin inhibitors and UST have also been published [73].

## 3. Predictors of Biologic Therapy Response

There are several studies trying to find predictors of biologic therapy response, mainly with anti-TNFs. We will briefly summarize those available in clinical practice.

### 3.1. Genetic Predictors of Response

Genetic predictors have the potential advantage of remaining unchanged over time. The identification of distinctive genetic profiles of nonresponder patients may lead to understanding the predominant active mechanism of inflammation in these patients and may help to find a fit treatment. Recent genome-wide association studies (GWASs) suggest that beyond a few genes with large effects on biologic response, there may be several single-nucleotide polymorphisms with modest effects. Many genes have been evaluated, especially TNF-related genes, but the overall results are poor and there are no good predictive factors for anti-TNF response [74,75]. However, findings from the PANTS study have relaunched the genetic predictors to the foreground. The PANTS study is a prospective observational United Kingdom (UK)-wide study that included 955 patients with active luminal CD treated with IFX and 655 patients treated with ADA. The only factor associated with primary nonresponse to anti-TNF was low drug concentration at week 14, mediated in part by immunogenicity. Immunogenicity was twice as common in IFX-treated than ADA-treated patients at week 54, and combination with thiopurines or methotrexate mitigates this risk [76]. Specifically, the variant HLA-DQA1*05, present in 40% of patients of European descent, seems to significantly increase the risk of developing antibodies against IFX and ADA, regardless of concomitant immunomodulators. Evaluating HLA-DQA1*05 before starting biologic treatment may guide the selection of biological drugs and the selection of patients who can benefit more from adding a thiopurine.

### 3.2. Patient Characteristics

Age and gender have been studied as possible predictors of biologic therapy response in IBD. Data from a study suggest that anti-TNFs may be cleared faster in men than women, which seems to have a greater impact in biologics for which the dose is not adjusted by weight [64]. However, there is not enough evidence supporting gender as a predictor of response for anti-TNFs, VDZ, or UST [75]. Concerning age, the evidence is contradictory and will be discussed later [75].

### 3.3. Inflammatory Markers (Albumin, C-Reactive Protein (CRP), Calprotectin)

An interesting pharmacokinetic study analyzed factors associated with a higher IFX clearance in IBD [77]. Patients with low albumin levels had a higher IFX clearance, and in this study, an association between low albumin and the risk of developing antibodies was suggested, perhaps due to an intermittent IFX exposure. Authors suggest that shortening the interval of infusions at induction could be the best strategy.

Data regarding the utility of CRP as a predictor of anti-TNF response are controversial. In UC, patients with lower levels of CRP seem to have a better response to IFX and ADA [78,79]; however, in other studies, there was no association [80,81]. In CD, there are many studies assessing an association between high levels of CRP and anti-TNF response [82,83,84], but others fail to find an association [8,85]. In a review article about this topic, the authors conclude that the predictive value of CRP in this context is possible in CD and controversial in UC [75]. The same authors did not find a significant association between calprotectin levels and anti-TNF or VDZ response. Concerning VDZ, the value of CRP as a predictor of response is also controversial. In UC, lower levels are associated with a better response, while in CD the evidence is inconclusive [86,87]. In the case of UST in CD, there is also no evidence suggesting the utility of CRP as a predictor of response [32,33].

## 4. Other Aspects to Consider before Choosing a Biologic

### 4.1. Age and Response 

Treatment of elderly patients with IBD can pose certain unique challenges. Firstly, advanced age is a risk factor for several comorbidities, including cardiovascular disease, diabetes, and cancer, which complicate the use of biological drugs. Secondly, the elderly population may be different in terms of absorption, distribution, and excretion of drugs compared with the younger age population [88]. Thirdly, there is often a mismatch between chronological and biological age. Not all “elderly patients” are the same, and it is important to distinguish between age and frailty [89]. Therefore, the use of biological treatment in elderly IBD patients requires a careful assessment of the efficacy and safety profile. Clinical trials often exclude or underrepresent elderly patients, and observational studies examining biologic drugs in this subpopulation have small sample sizes, so treatment decisions are usually based on extrapolated evidence.

The evidence supporting the efficacy of anti-TNF therapy in elderly patients is conflicting. Some studies showed lower response rates and lower persistence with therapy [90,91], but it was not apparent in other cohorts [92,93]. However, the evidence indicating that adverse events are more frequent in the elderly population is robust [94]. A meta-analysis that compared the safety of biological drugs (mostly anti-TNF drugs) across age groups for immune-mediated diseases showed that infections were more prevalent in elderly patients treated with biologics than in younger patients treated with the same therapies (OR 2.28) and in elderly patients not treated with biologics (OR 3.60). Malignancies were also more frequent in elderly patients treated with biologics than in younger users (OR 3.07) but not when compared to elderly controls. The analysis restricted to the six studies including IBD patients showed that older anti-TNF users had an elevated risk of infection (OR 3.48) and malignancy (OR 3.47) than younger users [95]. Regarding combination therapy, the European Crohn’s and Colitis Organisation (ECCO) recommends anti-TNF monotherapy over combination therapy in elderly patients. Although there are no specific studies, optimizing doses to reach adequate (or even higher) trough levels is not associated with an increased risk of adverse effects in this subpopulation [96].

Data about VDZ are limited, but GEMINI trials showed a similar efficacy and safety in all age subgroups [97]. Some studies have compared VDZ and anti-TNF in elderly IBD patients with controversial results; two studies did not show differences in the incidence of significant infections or efficacy, but another showed a reduction in infection-related hospitalization with VDZ [98,99,100]. There are no studies directly comparing the efficacy and safety of UST in elderly IBD patients, but the IM-UNITI trial reported similar rates of adverse events between UST and placebo across all age subgroups [101]. In two retrospective psoriasis studies (UST dose is lower in psoriasis than in IBD), an increased risk of adverse events was not observed in elderly patients [102,103]. Briefly, VDZ and UST may be prioritized in elderly patients, especially in frail elderly patients.

### 4.2. Comorbidities

Comorbidities in IBD patients may have a negative impact on the safety and effectiveness of biologics.

Obesity and overweight are increasingly common in IBD patients. Observational studies in rheumatology have reported a worse response to anti-TNF in obese patients [104]. Based on pharmacokinetic studies, this may be due to two main reasons: an increased volume of distribution with consequent low drug trough concentrations and higher systemic inflammatory burden due to obesity-induced low-grade inflammation, [105,106]. However, in IBD studies, obesity is not clearly associated with an impaired response to TNF inhibitors. Some studies have reported a higher response rate in IBD patients with lower weight, and others have reported opposite results [76,79,107]. A recent pooled data analysis of IFX-RCTs in IBD (ACCENT-1, SONIC, ACT-1, and ACT-2) showed that there was no association between obesity and the probability of clinical remission [108]. Evidence about the impact of obesity on UST or VDZ efficacy is scarce [109]. In psoriasis studies, increasing dose in patients with high weight (>100 kg) was associated with more effectiveness [110,111]. Therapeutic drug monitoring can be a very useful tool in obese patients.

Anti-TNF agents should not be used in patients with congestive heart failure (NYHA class III/IV) because they can worsen it. Conversely, there is no evidence of worsening heart failure using non-anti-TNF biologics [112]. In addition, it is known that anti-TNF therapy is not appropriate in a patient who has a demyelinating disease (e.g., optic neuritis or multiple sclerosis) because such treatment can worsen outcomes. Therefore, UST or VDZ may be prioritized in IBD patients with these comorbidities [113].

Finally, given that anti-TNFs seem to be associated with a higher risk of infections than UST or VDZ, the latter may be prioritized in patients at high risk of serious infections or with prior history of infections requiring hospitalization [113].

### 4.3. Presence of Extraintestinal Manifestations

When choosing a biologic, the presence of extraintestinal manifestations could cause some drugs to be preferred over others.

#### 4.3.1. Arthropathy

In general, in polyarthritis and axial arthropathy, IBD activity is independent of articular inflammation, while in peripheric monoarthritis, IBD activity is usually associated with articular inflammation. In the situation of a patient with IBD and axial arthritis, both active and with indication for biologic therapy, the drugs with more evidence are anti-TNFs. IFX and ADA can be used for CD and UC, but GOL is only effective in UC [114,115,116]. The dose should be the one generally used for IBD, which is higher than the one used only for arthropathy. VDZ seems to be ineffective for axial arthropathy, and there is also a lack of evidence supporting the use of UST in this context [117,118]. If the IBD is active and the patient also has peripheral arthritis, the best choice would also be anti-TNF therapy [119]. In patients with active arthropathy without IBD activity, it is mandatory to ensure IBD remission, for example, using calprotectin, endoscopy, or magnetic resonance imaging (MRI) [119]. In these cases, it is recommended to use anti-TNFs which have also demonstrated to be effective for the specific type of IBD (UC or CD) at rheumatologic dose [119]. If the arthropathy is inactive but a biologic therapy is necessary to control the IBD, we should choose it based on IBD algorithm, with anti-TNFs as first choice [113,119]. In all cases, if it is necessary to associate an immunomodulator, the preferred one should be methotrexate [120].

#### 4.3.2. Ocular Manifestations

In patients with ocular involvement (scleritis and uveitis), the control of IBD activity usually leads to ocular disease remission. If a biologic therapy is necessary, anti-TNFs are the first choice, with more evidence about ADA [121,122].

#### 4.3.3. Skin Manifestations

In patients with erythema nodosum and pyoderma gangrenosum, the IBD activity control is the best therapy; however, if a biologic is required, anti-TNFs are the preferred ones [123,124,125].

Some IBD patients can also be affected by psoriasis. Anti-TNFs, ADA and IFX, have been demonstrated to be effective for both indications (IBD and psoriasis) [126,127]; however, it should be noted that, in some patients, anti-TNFs can induce paradoxical psoriasis and make preexistent lesions worse. These lesions can improve with topical therapy and usually disappear when the drug is withdrawn. The swap of biologic therapy could be necessary [128]. UST is another safe and effective option in the treatment of IBD associated with psoriasis and, also, in the management of anti-TNF paradoxical psoriasis [129].

In patients with IBD, especially in CD, there is an increased risk of hidradenitis suppurativa. In this setting, the biologic therapy with more evidence is the anti-TNFs [130].

### 4.4. Patient Preferences

There are different routes of administration of the biologics, and this should be considered in order to improve patients´ adherence. For example, subcutaneous therapies could be preferred in patients who do not want to come to the hospital for infusions. On the other hand, in patients with a lack of adherence, intravenous infusions could be a better option, because we assure that the patient is taking the treatment. In some cases, industry-provided patient assistance programs could be useful too. There are also differences in subcutaneous formulations. For example, in ADA therapy, the citrate has been removed from the original Humira because it was associated with painful injection, and this strategy seems to increase the patients’ adherence [131].

In patients at reproductive age, the desire for pregnancy can also be important in selecting the biologic. Most data about biologic therapy and pregnancy comes from anti-TNFs, which have been demonstrated to be safe. In the case of VDZ and UST, there are fewer studies in pregnancy, but they are not associated with poor outcomes [132]. The AGA suggests, if possible, taking the last dose of the biologic in the third trimester according to its half-life, in order to minimize the fetal transmission of the drug as long as the remission is achieved [133].

### 4.5. Speed of Onset

The onset of action should also be considered. For example, the clinical improvement in patients taking VDZ appears from week 2 [134], especially in bio-naïve patients. Moreover, anti-TNFs are known for their fast onset of action, especially IFX, which has been demonstrated to be effective in acute severe UC [70,135]. There are also data in naïve patients with CD treated with ADA, assessing clinical improvement from day 4 of therapy [136]. Depending on the clinical situation, a fast onset of action could be necessary.

### 4.6. Costs and Availability

Since the introduction of the biologic therapy in IBD, the treatment-related costs have increased significantly [137]. So, the biologic therapy is an important part of IBD-care costs, and for that reason, many cost-effectiveness studies have been published recently.

#### 4.6.1. Cost-Effectiveness Studies of Biologic Therapy

When evaluating the costs of a therapy, we have to consider not only the cost of the drug itself, but also the need for hospitalization, intravenous infusions, consultant visits, complementary explorations, surgery, and adverse-event-related costs. In a UK study, the acquisition cost of VDZ (300 mg) in 2017 was GBP 1678.48, while that of IFX (100 mg) was GBP 419.62 [138]. However, in this study, considering all that has been previously mentioned, VDZ is a cost-effective therapy compared with anti-TNFs in moderate–severe UC. Nevertheless, in a Spanish study with a higher price for VDZ and a EUR 30000/quality-adjusted life year threshold, ADA would be cost-effective in 64% cases, IFX in 29.1%, GOL in 7.1%, and VDZ in 0.5% in bio-naïve patients [139].

In an American study, the cost-effectiveness of VDZ in UC was found to depend on the type of anti-TNF most frequently prescribed as first-line therapy [140]. In a Polish cohort of bio-naïve UC patients, comparing with standard care, the most favorable increment in cost-effectiveness was found for the treatment with IFX and VDZ [141]. For moderate–severe CD, in a US study in bio-naïve patients, IFX was the most cost-effective biologic, followed by ADA and UST [142].

Regarding the price of therapies, in some countries, especially those with health insurance policies, the coverage included should also be taken into account when choosing between two equally effective therapies.

#### 4.6.2. Biosimilars

The development of biosimilars seems to decrease the biologic-related costs [137,143,144], and its use in bio-naïve patients is supported by the main scientific associations, but with low quality of evidence [145,146,147]. The noninferiority strategy of switching IFX for a biosimilar has also been proven [148]. In a Hungarian study, the rates of clinical remission at week 14 with biosimilar IFX were higher in anti-TNF-naïve compared with previous exposure to originator IFX (in CD 60.9% vs. 35.7% and in UC 65.1% vs. 33.3%, respectively; *p* < 0.005); nevertheless this difference was not statistically significant at week 30 for both CD and UC [149].

In the PROSIT-BIO cohort, including anti-TNF-naïve patients, patients with previous exposure, and patients with a switch to a biosimilar, the efficacy of IFX biosimilar seems to be the same as the original [150]. In a French study including 5050 CD IFX-naïve patients, there was equivalence in terms of death, surgery, hospitalization, and change of biologic therapy between IFX biosimilar and original [151]. The efficacy in UC IFX-naïve patients does not differ from biosimilar and the original [152].

In a Sicilian study of IBD patients treated with ADA biosimilar, including anti-TNF-naïve and previous exposure patients, efficacy and safety of ADA biosimilar did not differ from the original [153]. The efficacy of ADA biosimilar has also been demonstrated in other studies including UC and CD [154,155].

## 5. Positioning Biologic Therapies in the Management of IBD

### 5.1. In Crohn’s Disease

In patients with moderate–severe CD, we recommend both IFX and ADA as first-line therapies. IFX may be preferred over ADA in patients with higher inflammatory burden, perianal disease, or fistulizing pattern.

In patients with moderate–severe CD with high disease severity, who have relative or absolute contraindications to anti-TNF drugs (e.g., demyelinating diseases, heart failure, multiple serious infections), we prefer UST monotherapy as first-line therapy. However, in fragile patients with moderate CD and higher risk of treatment-related complications or in treatment with other high-risk immunosuppressive therapies, VDZ monotherapy could be preferred.

### 5.2. In Ulcerative Colitis

In moderate–severe UC, we recommend both IFX and VDZ as first-line therapies, and the keys to guide this decision are the costs, the risk of adverse events, and the probability of developing secondary loss of response. The most important factor would probably be the price, since the availability of IFX biosimilar significantly decreases the costs when compared with VDZ and the two of them are equally effective. In fragile patients or those with higher risk of infections, VDZ could be preferred. In those patients who desire subcutaneous treatment, IFX and VDZ are the best choices if available, but if not, GOL and UST are good alternatives.

In the acute ulcerative colitis setting, the only biologic we can recommend is IFX, based on the current evidence.

## 6. Conclusions

The number of biologic therapies available for IBD is increasing, and there are many studies assessing the efficacy and safety of each therapy separately; however, the lack of head-to-head trials makes it difficult to choose the best therapy for a specific patient among the options available. Data from meta-analysis and real-world experience are useful, but individual characteristics such as age, patient preferences, and comorbidities, as well as costs, must be considered. Table 3 summarizes our recommendations, based on all that has been mentioned above, about the most appropriate biologic therapy in particular situations. It should be noticed that small molecules are not discussed in this review, but tofacitinib and other drugs that are going to be available in the near future (e.g., filgotinib, upadacitinb, etrasimod) will probably change the decision algorithm.

## Figures and Tables

**Table 1:** Real-world studies comparing effectiveness between biologicals in anti-TNF-naïve CD patients.

Authors (Year)	Biological Drugs	Patients	Sample Size	Follow-Up	Main Results	Conclusion
Kestens et al. (2013) [7]	IFX versus ADA	Anti-TNF naïve	200 patients (100 IFX, 100 ADA)	1 and 2 years	*Steroid-free clinical response:* IFX (at 1 and 2 years): 65% and 49% ADA (at 1 and 2 years): 62% and 41%	No difference between IFX and ADA
Narula et al. (2016) [8]	IFX versus ADA	Anti-TNF naïve	362 patients (251 IFX, 111 ADA)	1 year	*Steroid-free remission at 12 months:* IFX: 44.3% ADA: 53.7%	No difference between IFX and ADA
Cosnes et al. (2016) [9]	IFX versus ADA	Anti-TNF naïve	906 patients 1284 therapeutic exposures to ADA (*n* = 521) or IFX (*n* = 763)	2 years	*Response rate at 6 months and at 2 years:* IFX mono: 72% and 45% IFX combo with immunomodulator: 84% and 68% ADA mono: 64% and 44% ADA combo with immunomodulator: 86% and 70%	No difference between IFX and ADA Combination therapy superior to monotherapy
Macaluso et al. (2019) [10]	IFX versus ADA	Anti-TNF naïve and experienced	632 patients 735 total treatments	1 year	*Clinical benefit (steroid-free remission or clinical response) in naïve patients at 12 weeks and 1 year:* IFX: 77.6% and 64.5% ADA: 81.8% and 69.2%	No difference between IFX and ADA Lower response rates among anti-TNF experienced compared to naïve
Osterman et al. (2014) [11]	IFX versus ADA	Anti-TNF naïve	2330 patients (1459 IFX, 871 ADA)	26 weeks	*Persistence on therapy:* IFX 49%, ADA 47%	No difference between IFX and ADA
Singh et al. (2018) [12]	IFX versus ADA	Anti-TNF naïve	827 patients (512 IFX, 315 ADA)	2 years	*CD-related hospitalization*: HR 0.81 (95% CI 0.55–1.20) *Major abdominal surgery:* HR 1.24 (0.66–2.33) *Serious infections*: HR 1.06 (0.26–4.21)	No difference between IFX and ADA in efficacy and safety
Macaluso et al. (2021) [13]	VDZ versus ADA	Anti-TNF naïve and experienced	585 treatments (277 VDZ, 308 ADA)	56 weeks	*Clinical response (week 52): ADA: 69.1% and VDZ: 64.3%* *Mucosa healing: ADA: 33.8% and VDZ: 31.8%*	No differences between VDZ and ADA
Bohm et al. (2020) [14]	VDZ versus anti-TNFs (IFX, ADA, and CTZ)	Anti-TNF naïve and experienced	1266 patients (659VDZ)	1 year	*Steroid-free clinical remission*: HR 1.250, 95% CI 0.677–2.310 *Endoscopic remission*: HR 0.827, 95% CI 0.595–1.151 *Noninfectious serious adverse events*: OR 0.072, 95% CI 0.012–0.242 *Serious infections:* OR 1.183, 95% CI 0.786–1.795	Lower risk of noninfectious serious adverse events, but not serious infections, with VDZ vs. anti-TNF No significant difference for achieving disease remission (clinical and endoscopic)

Abbreviations: Infliximab (IFX), adalimumab (ADA), vedolizumab (VDZ), certolizumab (CTZ), Crohn´s disease (CD), hazard ratio (HR), confidence interval (CI).

**Table 2:** Real-world studies comparing effectiveness between biologicals in anti-TNF-naïve UC patients.

Authors (Year)	Biological Drugs	Patients	Sample Size	Follow-Up	Main Results	Conclusion
Shing et al. (2017) [64]	IFX versus ADA	Anti-TNF naïve	171 IFX, 104 ADA (propensity-score-matched cohort)		*All-cause hospitalization rate ADA vs. IFX:* HR 1.84 (95% CI 1.18–2.85) *UC-related hospitalization rate ADA vs. IFX:* HR 1.71 (95% CI 0.95–3.07)	Higher risk of hospitalization with ADA than IFX
Bressler et al. (2021) [65]	Anti-TNFs versus VDZ	Anti-TNF naïve	604 UC patients (138 IFX, 62 ADA, 24 GOL, 380 VDZ)	24 months	*Clinical response:* anti-TNFs 86.2% vs. *VDZ* 88.3%; *p* = 0.64 *Clinical remission:* anti-TNFs 48.6% vs. *VDZ* 65.9%; *p* = 0.09 *Mucosal healing:* anti-TNFs 80.6% vs. *VDZ* 86.6%; *p* = 0.66	No difference between anti-TNFs and VDZ
Helwing et al. (2020) [66]	Anti-TNFs versus VDZ	46.5% anti-TNF naïve	133 UC (57 anti-TNFs, 76 VDZ)	26 weeks	*Clinical remission in bio-naïve:* anti-TNFs 31.5% vs. VDZ 50.1%; *p* = 0.15	Anti-TNFs and VDZ are effective
Patel et al. (2019) [67]	VDZ versus IFX	Anti-TNF naïve	1721 (542 VDZ, 1179 anti-TNFs)	24 months	*Treatment persistence rates at 12 months: VDZ* 84.5% vs. anti-TNFs 77.5%; *p* = 0.006 *Treatment persistence rates at 24 months: VDZ* 77.6% vs. anti-TNFs 64.6%; *p* = 0.0005	VDZ is superior to anti-TNFs in long-term effectiveness
Allamneni et al. (2018) [68]	VDZ versus IFX	42.4% anti-TNF naïve	59 patients (32 VDZ, 27 IFX)	Until assessment for clinical response	*Clinical response rates in bio-naïve:* VDZ 6.74/100 person-weeks vs. IFX 6.48/100 person-weeks	No difference between VDZ and IFX

Abbreviations: Infliximab (IFX), adalimumab (ADA), vedolizumab (VDZ), golimumab (GOL), ulcerative colitis (UC), hazard ratio (HR), confidence interval (CI).

**Table 3:** Authors’ recommendations for biologic choice in different situations.

Situation	Recommendation
Luminal CD	IFX and ADA seem to be the best options UST seems useful too VDZ seems useful too
Fistulizing CD	IFX seems to be the best option ADA, UST, and VDZ seem useful too
Acute severe UC	IFX
Moderate–severe UC	IFX and VDZ seem to be the best options GOL and UST are useful too ADA seems to be less effective
HLA-DQA1*05 (patients are at risk of secondary loss of response due to immunogenicity)	Use anti-TNF combination therapy or other molecules (in patients at risk of adverse events due to combination therapy, other biologics could be preferred)
Pregnancy desire	The drugs with more evidence are anti-TNFs UST and VDZ also seem to be safe
Elderly patients	UST and VDZ could be preferred, especially if we want to avoid combination therapy with anti-TNFs
Arthropathy • IBD active and axial/peripheral inflammation • IBD inactive and active arthropathy • IBD active and inactive arthropathy	UC: IFX, ADA, GOL CD: IFX, ADA Anti-TNFs also effective for IBD (rheumatologic dose) Select biologic according to IBD algorithms, suggest anti-TNFs as first choice
Episcleritis or uveitis	Anti-TNFs (more evidence with ADA)
Erythema nodosum and pyoderma gangrenosum	Anti-TNFs
Psoriasis associated	Anti-TNFs or UST
Hidradenitis suppurativa	Anti-TNFs
Patients’ route of administration preference	• Subcutaneous: UC (in order of preference: GOL, UST, ADA)*, CD (ADA, UST)* • Intravenous: IFX, VDZ*
Low adherence	Biologic with intravenous administration could be preferred
Low resources	Anti-TNF biosimilars could be preferred

* Subcutaneous administration of VDZ and IFX are also available in many countries. Abbreviations: Crohn´s disease (CD), ulcerative colitis (UC), inflammatory bowel disease (IBD), Infliximab (IFX), adalimumab (ADA), golimumab (GOL), vedolizumab (VDZ), ustekinumab (UST).

## Data Availability

No new data were created in this study.

## Supplementary Materials

The following are available online at https://www.mdpi.com/article/10.3390/jcm11030829/s1, Table S1: Randomized placebo-controlled trials of biologic therapy in moderate–severe ulcerative colitis [156,157,158,159,160].
