# Supplementary material for: How to Choose the Biologic Therapy in a Bio-naïve Patient with Inflammatory Bowel Disease
Source: J Clin Med. 2022 Feb 4;11(3):829. doi: 10.3390/jcm11030829 (PMC8837085; doi:10.3390/jcm11030829)
Supplement: Supplementary file 1 [file jcm-11-00829-s001.zip › jcm-1523710-supplementary.pdf]

Table S1. Randomized placebo-controlled trials of biologic therapy in moderate-severe ulcerative colitis**.**

Study (year)	Biological drug	Patients	Sample size	Follow up	Main results.	Conclusion
ACT 1 and 2 (2005) [156]	IFX	Anti-TNFs naïve	364 patients	ACT 1: 54 weeks<br><br>ACT 2: 30 weeks	*Clinical response at week 8:*<br>ACT 1: 69% IFX 5 mg vs 61% IFX 10 mg vs 37% placebo; p <0.001<br>ACT 2: 64% IFX 5 mg vs 69% IFX 10 mg vs 29% placebo; p <0.001<br><br>*Clinical response at week 54:*<br>ACT 1: 45% IFX 5 mg vs 44% IFX 10 mg vs 20% placebo; p <0.001	IFX is superior to placebo
ULTRA 1 (2011) [79]<br><br>ULTRA 2 (2012) [157]	ADA	Anti-TNFs naïve<br><br>59.7% anti-TNFs naïve	390 patients<br><br>494 patients	ULTRA 1: 8 weeks<br><br>ULTRA 2: 52 weeks	*Clinical remission at week 8:* 18.5% ADA vs 9.2% placebo; p =0.031<br>*Clinical remission in bio- naïve at week 8:* 21.3% ADA vs 11% placebo;<br>p =0.017<br>*Clinical remission in bio- naïve at week 52:* 22% ADA vs 12.4% placebo;<br>p =0.029	ADA is superior to placebo
PURSUIT (2014) [158,159]	GOL	Anti-TNFs naïve	774 patients (phase 3)<br><br>464 patients	6 weeks (induction)<br><br>54 weeks (maintenance)	*Clinical response at week 6*: 51.8% GOL 200/100 mg vs 29.7% placebo; p <0.0001<br><br>*Clinical response at week 54*: 47.0% GOL 50 mg vs 49.7% GOL 100 mg vs 31.2% placebo; p =0.010 and p <0.001<br>*Clinical remission and mucosal healing at week 54*: 41.7% GOL 50 mg vs 42.4% GOL 100 mg vs 26.6% placebo; p =0.002	GOL is superior to placebo
GEMINI (2013) [160]	VDZ	51.8% anti-TNFs naïve	895 patients	52 weeks	*Clinical response at week 6:* 47.1% VDZ vs 25.5% placebo (p<0.001)<br>*Clinical remission at week 52:* 41.8% VDZ vs 15.9% placebo; p<0.001	VDZ is superior to placebo
UNIFI (2019) [47]	UST	46.1% anti-TNFs naïve	961 patients	44 weeks	*Clinical remission at week 8:* 15.5% UST vs 5.3% placebo; p<0.001<br>*Clinical remission at week 44:* 38.4% UST/12 weeks vs 43.8% UST/8weeks vs 24% placebo; p=0.002 and p<0.001	UST is superior to placebo
